# Modelling daily plant growth response to environmental conditions in Chinese solar greenhouse using Bayesian neural network

**DOI:** 10.1038/s41598-023-30846-y

**Published:** 2023-03-16

**Authors:** Gadelhag Mohmed, Xanthea Heynes, Abdallah Naser, Weituo Sun, Katherine Hardy, Steven Grundy, Chungui Lu

**Affiliations:** 1 School of Animal, Rural and Environmental Sciences, Nottingham Trent University, Brackenhurst Campus, Nottingham, NG25 0QF UK; 2 Department of Computer Science, Nottingham Trent University, Clifton Campus, Nottingham, NG11 8NS UK; 3 Intelligent Equipment Research Centre, Beijing Academy of Agriculture and Forestry Sciences, Beijing, 100097.3 China

**Keywords:** Computer science, Scientific data, Plant sciences

## Abstract

Understanding how plants respond to environmental conditions such as temperature, CO_2_, humidity, and light radiation is essential for plant growth. This paper proposes an Artificial Neural Network (ANN) model to predict plant response to environmental conditions to enhance crop production systems that improve plant performance and resource use efficiency (e.g. light, fertiliser and water) in a Chinese Solar Greenhouse. Comprehensive data collection has been conducted in a greenhouse environment to validate the proposed prediction model. Specifically, the data has been collected from the CSG in warm and cold weather. This paper confirms that CSG’s passive insulation and heating system was effective in providing adequate protection during the winter. In particular, the CSG average indoor temperature was 18 $^{\circ }$C higher than the outdoor temperature. The difference in environmental conditions led to a yield of 320.8g per head in the winter after 60 growing days compared to 258.9g in the spring experiment after just 35 days. Three different architectures of Bayesian Neural Networks (BNN) models have been evaluated to predict plant response to environmental conditions. The results show that the BNN network is accurate in modelling and predicting crop performance.

## Introduction

Climate change is the biggest challenge to global food security. Protected cultivation can protect crops from extreme weather conditions, reduce the incidence of pests and diseases, and ensure that food is provided all year round. Globally, using a greenhouse environment is the most popular way to produce horticultural crops, with an estimated 496,800 hectares (ha) in 2019, with total production worth 20 billion US dollars in 2020^1^. China has become the world’s largest economy in protected horticulture, with 3.3 million ha (polytunnels included) and a total output of 1.43 trillion Chinese Yuan in 2019, and after polytunnels (45%), Chinese solar greenhouses (CSG) (30.5%) are the second most popular choice of greenhouse structure across China (Institute of Protected Agriculture AoAPaE, 2020). However, in cooler climates, the heating energy demand in a commercial greenhouse is responsible for 65-85% of total the greenhouse energy demand^2^.

**Figure 1:**
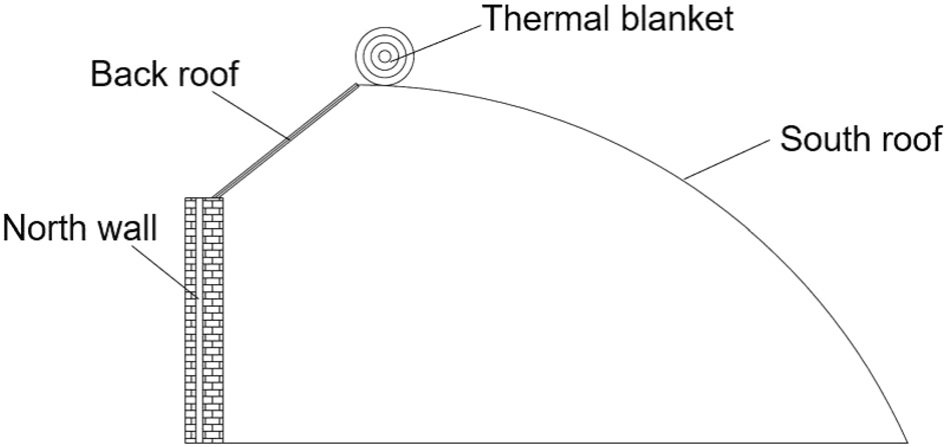
Cross-section of the Chinese solar greenhouse (CSG). The envelope consists of a north wall, side walls, and a back roof. The south roof is covered by transparent material.

CSGs employ a passive thermal recycling system to reduce the energy consumption needed through active heating, and external meteorological factors and control mechanisms determine the internal microclimate of a greenhouse (e.g., ventilation openings, exhaust fans, heaters, and evaporative cooling systems)^3^. However, they have significant structural differences compared to greenhouses in the Netherlands, Israel, and Spain^4,5^ regarding the cover, envelope and structure. A CSG has three thermal storage walls along the structure’s north, east, and west sides. The north wall, a core feature of the CSG, plays an essential role in thermal storage, heat preservation, and heat insulation^6^. The cambered south roof, north wall, and thermal blanket enable the CSG to perform well on daylight access, heat storage and insulation, as shown in Fig. 1. During the day, the greenhouse captures heat from the sun, storing it within the thermal mass of the walls, which is then released, at night, as a passive heating source. During the night, an insulating sheet closes over the transparent plastic sheet to reduce heat loss from the greenhouse. This passive solar heating strategy employed by CSGs enables huge energy savings compared to the heating required to heat a glass greenhouse. A study in Manitoba, Canada, showed that the supplemental energy required to maintain temperatures above 10 $^{\circ }$C at all times was 43 times less for the CSG compared to a glass greenhouse^7^.

Until recently, many approaches have been taken to optimise microclimate control performance and have considered the following environmental factors individually; light, temperature, humidity, ambient CO_2_ concentration, soil type, water, and nutrient availability. However advancements in environmental sensor technology which can record real-time fluctuations in microclimate, combined with data-driven machine learning approaches, offer the potential to resolve the highly complex relationship between these numerous factors which influence plant growth and development. For example, cultivation season can significantly impact plant growth and development, even when using an indoor greenhouse environment. Reduced light intensity and photoperiod experienced during winter reduces the physiological responses of plants (i.e., rate of photosynthesis and stomatal conductance), which negatively affects overall biomass yield, nutritional value, and is also attributed to increased nitrate content^8,9^. In this scenario, sensor technology could effectively record these real-time fluctuations, highlighting the requirement for an appropriate adaptation to improve the growing conditions of the CSG. Temperature, relative air humidity and CO_2_ concentration regulation in greenhouse environments must also be carefully considered. Whilst evaporative systems can be controlled by opening/closing the roof windows, this could adversely cause fluctuations in vapour pressure deficit (VPD)^10^, reducing a plant’s net CO_2_ assimilation rate^11^. A recent study analysed the plant growth characteristics of greenhouse lettuce grown under drastically fluctuating VPD conditions (1.63 kPa for 6 min and 0.63 for 3 min) and moderately fluctuating VPD conditions (1.32 kPa for 7 min and 0.86 kPa for 3 min), concluding dry shoot weight and leaf area was 15 and 29% lower in lettuce grown under drastically fluctuating conditions^10^. Temperature is also critically important environmental variable for maximising crop yield and productivity. The relationship of temperature and crop development often shows a sigmoidal relationship, where growth and development cease below a critical temperature threshold at both extremes, but a linear positive correlation exists between the two extreme thresholds. Regression analysis of time to harvest of field grown Romain Lettuce in South Carolina grown over multiple years identified that for every 1 $^\circ$C decrease in growing season mean (GSM) minimum or maximum temperatures from the optimum values, days to harvest increased by 5 days with a 5 $^\circ$C increase in GSM min or GSM max temps, total days to harvest increased 50%^12^. By dividing complex environmental data into elements, their effects on crop growth could be quantified, enabling an accurate prediction of the impact of fluctuating environmental conditions on plant growth.

The development of predictive models for plant growth in protected horticulture will allow for the optimisation of microclimate control strategies to maximise crop yield while reducing energy consumption. Currently, most CSG climate solutions are controlled manually based on experience, resulting in poor performance on greenhouse production. Many research works have been conducted to analyse and predict plant growth performance using many different Artificial Intelligence (AI) approaches to predict the environmental conditions, mainly ambient temperature^13^. Hence, there is currently no data model for lettuce growth in CSGs which incorporates climate management control including the effects of air temperature, humidity, CO_2_ concentration, and radiation under CSG scenarios, where environmental conditions are usually not within the optimal range for crop growth and development due to the limited climate control ability. Currently, AI is mainly employed for indoor and outdoor agriculture to enhance plant productivity by finding the most suited conditions for plant growth in terms of soil management, crop management, weed management and disease management. For indoor agriculture, the main concept of using AI is its flexibility, high performance, accuracy, and cost-effectiveness. Regulation of environmental components in greenhouses is crucial for better plant growth and many studies support that this can be achieved through employing Artificial Intelligence (AI) systems over manual control methods^14,15^. DL has also been applied to greenhouse yield prediction, although this has predominantly focused on tomato crops. A Dynamic Artificial Neural Network (DANN) is implemented in^16^, to predict tomato yield using phenotypic parameters including CO_2_ fixation, transpiration, as well as environmental parameters such as solar radiation and past yield. While CO_2_ fixation was found to be the most important variable, a high degree of predictive accuracy (R=0.917) was found with external parameters alone. Alhnaity et al (2019) evaluated several Machine Learning (ML) and Deep Learning (DL) techniques to achieve high predication accuracy in plant yield and growth within greenhouse environments using two different plants; ficus and tomato. The study specifically focused on ficus growth and the variation in stem diameter throughout their development, and tomato yield measurements, in combination with environmental measurements^17^. Recent research work by Gong et al. (2021) exhibited that DL based on a LSTM model achieved high prediction accuracy for both problems, outperforming classical ML approaches. This was achieved through applying artificial neural networks to predict tomato yield in a greenhouse environment based on historical yield and environmental data. Based on statistical analysis of the RSME, deep learning approaches outperformed classical models, with a combined tempo- ral convolutional network (TCN) with recurrent neural network (RNN) model providing the most accurate tomato yield predictions^18^.

These studies provide examples of how the development of predictive models for plant growth in protected horticulture will allow for the optimisation of microclimate control strategies to maximise crop yield while reducing energy consumption. Model predictive control has a large potential to provide higher control efficiency. As the basis, a crop model responding to greenhouse climate is needed. DL has demonstrated how it can be used as a powerful tool for yield prediction, however its application in a greenhouse environment has focused primarily on tomatoes grown in European style greenhouses. These approaches are utilised for dealing with the randomness and complexity of agricultural data including data that obtained from CSG. To give a better understanding of the data-driven approaches proposed for agricultural data processing, this paper grouped the data-driven methods into three main groups: Deterministic Methods, Stochastic Methods, and Machine Learning (ML) methods^19^. In this paper, an ML method, called BNN, has been utilised to enhance the crop productivity through predicting plant response to environmental conditions in CSG. The rationale behind using an ML approach, in particular, BNN is to overcome the limitations of stochastic and deterministic methods, e.g., deterministic methods can not deal with high random distribution data, which likely to accrue in agricultural environment^20^. While stochastic method, e.g., Markov Chain Model, Hidden Markov Model (HMM), and entropy, is a promising approach in agricultural applications due to its computational and time efficiency^21^. The potential randomness measure of agricultural data can be analogous plant growth response.

The main aim of this study is to generate a predictive model to understand the effect of environmental conditions on lettuce yield in CSG’s to enhance plant performance and resource-use efficiency. This will be achieved through the following objectives: (1) collect temperature, light, CO_2_ and humidity data across a warm and cold season to train the ANN structures, (2) determine the most effective structure based on the data generated from these environmental parameters.

This section has introduced the study and provided an overview of the related works. The rest of this research paper is organised as follows; the experimental setup is presented in section Experimental setup, including data collection, data pre-processing and the proposed models. The results from the proposed Bayesian neural network-based model are presented and discussed in section Results and discussion including an evaluation of the following environmental benefits: temperature, light, CO_2_, and humidity. This section also includes a discussion on the evaluation of seasonal difference in plant performance and modelling daily plant growth response to environmental conditions using BNN. Finally, a conclusion is drawn in section Conclusion with suggestions for future work provided.

**Figure 2:**
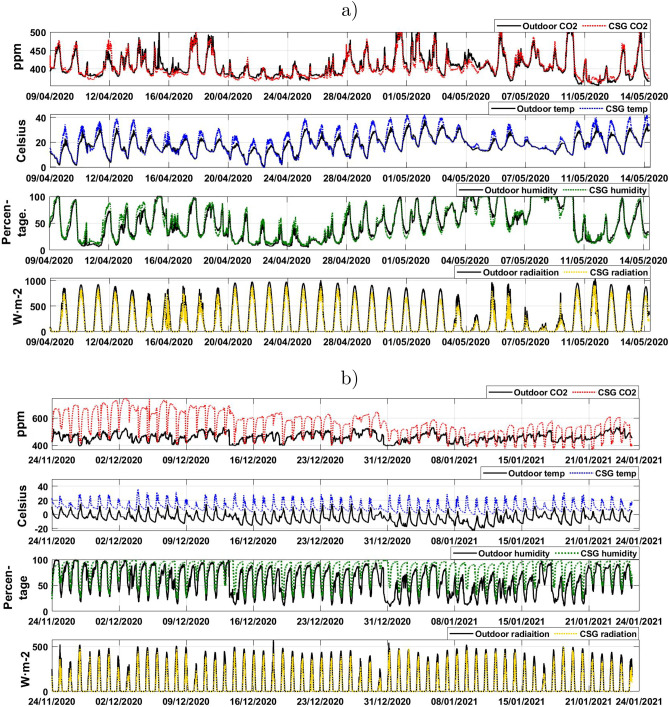
The collected data representing the environmental variation across the “warm” late spring and “cold” early winter experiments, (**a**) the collected data during experiment 1 for 35 days, (**b**) the collected data during experiment 2 for 60 days.

## Experimental setup

To evaluate the performance of the proposed approach, two different datasets were collected from a CSG during warm and cold seasons. In particular, the BNN, explained previously, is employed with the collected datasets to observe the effect of different environmental conditions on lettuce growth. Specifically, the lettuce cultivar (41–27) Tiberius RZ (produced by RIJK ZWAAN, the Netherlands) was selected for experimental trials. In the following sections, the collected datasets are explained in detail, as well as the BNN based model for modelling lettuce growth, based on the datasets collected from a Chinese Solar Greenhouse.

### Data collection

The data used in this research was collected from a CSG located in Beijing, China. The CSG is oriented east-west, consisting of the north wall, side walls, south roof, and back roof, as well as the two controllable structural components of thermal blankets and vents. No climate conditioning equipment was used during the experiments. Water and fertiliser management, as well as pest control, were assumed to be ideal.

Environmental conditions including temperature, CO_2_, relative humidity and light radiation were measured inside and outside of the CSG using a complex sensor module. Five temperature, humidity, and CO_2_ sensors and three radiation sensors were placed at different locations throughout the CSG. 6–18 lettuce plants were randomly harvested for each sample. When conducting the lettuce experiments during warm and cold seasons, two different datasets were collected to comparatively measure the indoor and outdoor environmental conditions of the CSG whilst simultaneously collecting plant response data for the lettuce plants.

The first dataset was collected during a warm season (April 9 to May 14, 2020). The CSG used for the warm weather experiment has a floor area of approximately 577 m$^{2}$, with a width of 7.4m and a length of 78m. The lettuce seedlings were transplanted to soil inside the experimental CSG when they had 11 fully developed leaves. The plant density is 5.30 plants/m$^{2}$ (floor area).

Following this, the second dataset was collected during a cold season, from November 24, 2020 to January 23, 2021. The CSG used for the cold weather experiment has a floor area of approximately 686 m$^{2}$, with a width of 7.95m and a length of 86.3m. The lettuce seedlings were transplanted to soil inside the experimental CSG when they had 5 fully developed leaves. The plant density 4.91 plants/m$^{2}$ (floor area).

The sensors measured environmental parameters every 5 minutes. The real-time of measuring the environmental conditions were also used for training the model. The main focus of this paper was to collect indoor data from a Chinese Solar Greenhouse and lettuce plant response data through measuring Shoot fresh weight, Root fresh weight, Shoot dry weight, Root dry weight and leaf area, in accordance with the measured environmental conditions. Table 1, shows the average of each environmental parameter measured indoor and outdoor of the CSG. To improve the training efficiency of the BNN, data normalisation and augmentation techniques were used with the collected environmental conditions data. 10082 and 17282 samples were measured for each indoor and outdoor parameter during warm and cold seasons, respectively. A total of 27,364 data points for each environmental parameter was collected from CSG during both the seasons.

**Table 1:** Information about the collected datasets including measured parameters, measuring unit and average for each measured parameter during the experiment period. EX1 data is the dataset collected during the warm season for 35 days. EX2 data is the dataset collected during the cold season for 60 days.

Measuredparameter	Ave temperature (C$^\circ$)		Ave CO_2_ (ppm)		Ave RelativeHumidity (%)		Ave Light Rad-iation (W m$^{-2}$)		Ave Wind speed (m s$^{-1}$)	
	EX1	EX2	EX1	EX2	EX1	EX2	EX1	EX2	EX1	EX2
Inside CSG	22.96	10.9	405.12	559.2	51.30	81.75	179.5	63.88	–	–
Outside CSG	17.2	$-$ 7.11	406.5	457.7	49.5	58.7	244.1	100.47	1.59	0.78

### Data pre-processing

BNN has been trained using both of the collected datasets illustrated in Fig. 2 and Table 1. The datasets represent both indoor and outdoor environmental conditions (temperature, CO_2_, relative humidity and light radiation), in addition to the daily yield measurements and growth rate (Shoot fresh weight (g), Shoot dry weight (g), Root fresh weight (g), Root dry weight (g) and leaf area cm${^2}$. The recorded data representing the environmental conditions were measured every 5 minutes, followed by being averaged on an hourly/daily basis. Simultaneously, the yield measurement growth data was recorded every 5 days. To deal with these data characteristics, the data augmentation technique was performed, through interpolation of days’ data, resulting in daily data measurements. An hourly average for the environmental parameters was also performed to achieve a similar daily representation that matched the yield measuring observations. Moreover, to deal with the missing information in the obtained dataset, the missing observations data were replaced by the moving average interpolation of the latest neighbouring time series data.

The response of lettuce growth to changes in environmental conditions can be categorised as a long response that can be identified on a daily basis. Therefore, resampling or aggregation of time series data is applied. This includes data being resampled on a daily basis using the averaging procedure to identify the growth response and the daily growth rate.

**Figure 3:**
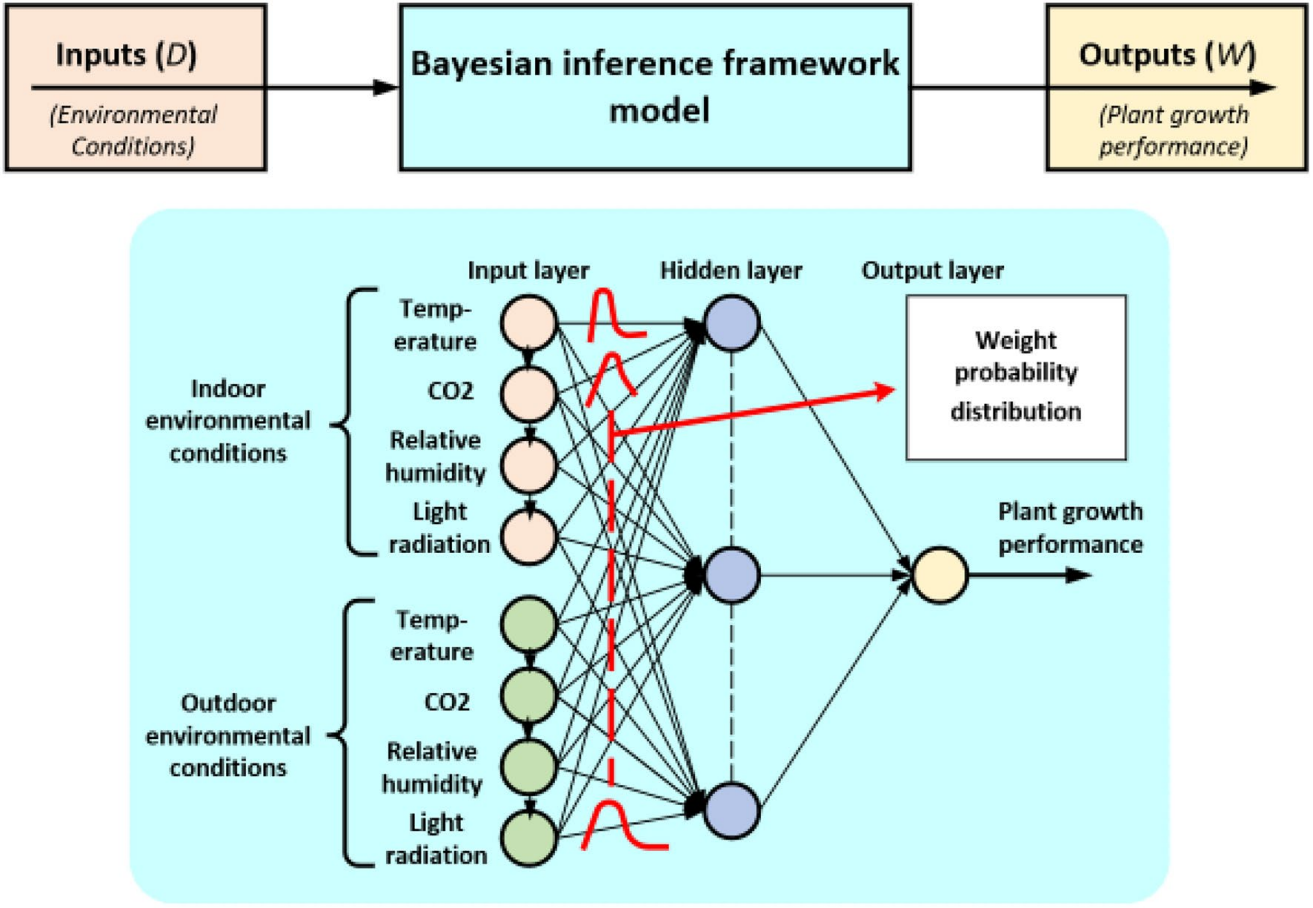
A simple block diagram of the Bayesian inference framework for updating probability distribution over the weights with multi-inputs and single output. Back-propagation in neural network structure for modelling lettuce growth is used in this framework. The network has three layers; input layer for the environmental conditions inside and outside of the CSG, one hidden layer and one output layer for the plant growth.

**Figure 4:**
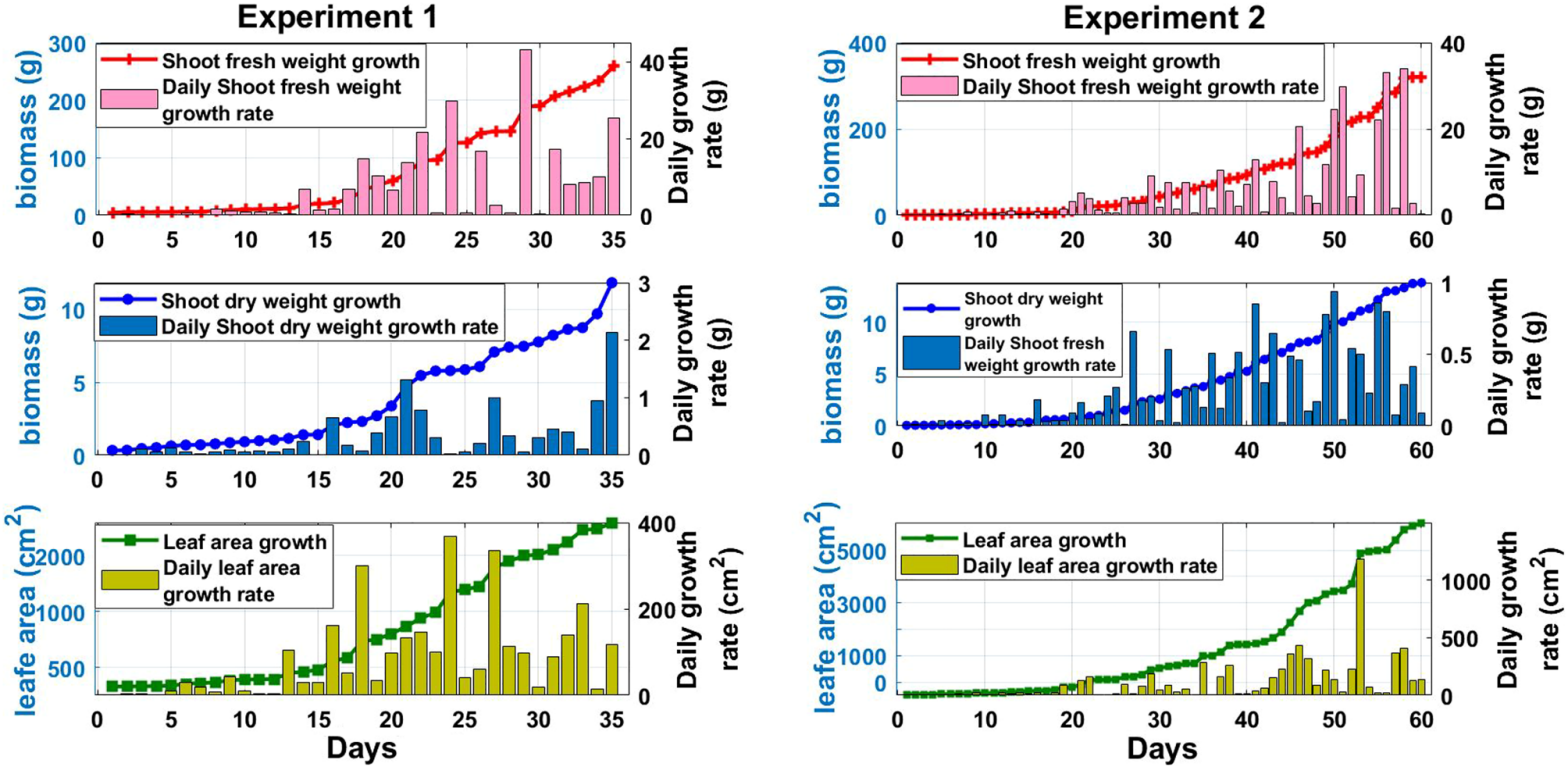
The overall growth performance and the daily growth rate responses of lettuce dry and fresh weights and leave area during experiment 1 and 2.

### Bayesian neural network-based model

In this study, Bayesian Neural Networks (BNNs)^22^ have been used for modelling and predicting lettuce plant growth in CSG based on the back-propagation algorithm^23,24^. The primary aim of the utilised network is to find the relationship between the network inputs (temperature, CO_2_, relative humidity, day and light radiation) and the network outputs (the biomass measurements (dry and fresh weight) for the plant shoot, root matter, and leaf area). Various neuron numbers in the hidden layer have been examined, including, 10, 20 and 25 neurons.

Figure 3 shows the architecture of the proposed approach using Bayesian neural network for modelling and predicting the lettuce growth in CSG. The BNN structure, used in this study, consists of three layers: input, hidden, and output layers. To obtain a high performance of modelling and lettuce growth , a feedback loop procedure was applied to produce time-series historical data for the input and output datasets. This was achieved by using a time delay operator with the inputs and outputs during the training mode.

To increase the ability of the designed BNN for modelling lettuce growth in the CSG with sufficient accuracy, the two collected datasets representing the warm and cold seasons were combined together and randomly split into three independent subsets: training, validation, and testing. The training dataset is the sample of data used for learning the BNN algorithm to fit the model. The validation dataset is used during the training mode consisting of the trained model and to regularise the early stopping training iterations to prevent the issue of model overfitting when generalisation was not improving^25^. The testing dataset is used to test the model after the training mode is done to evaluate the model performance. This means that the training and validation dataset *D* is (70%), and the testing set *W* is (30%). The training and validation datasets include $D_x$ and $D_y$ for the training input parameters and training labels, respectively.

As the structure of the used BNN model is significant in the performance of the model, the model parameters, consisting of the prior distribution, the likelihood function and the number of neurons in the hidden layer, needed to be determined. Therefore, the model parameters and the achieved results are determined using a Mean Absolute Error (MAE) for comparing the different used networks to determine the optimal structure.

## Results and discussion

Real-time environmental data was collected from indoor and outdoor of CSGs during two experiments performed in a warm season and cold season, and plant performance was measured throughout. This data provides insight into the performance of the CSG and has also been used to develop a predictive crop growth model using BNN from the environmental and plant performance data. It is becoming increasingly prominent that AI and human–machine learning can improve our understanding of how input features can influence behaviours in volatile environments; simultaneously improving prediction accuracy and reliability, which are important components of Agriculture^26^. Based on observations from the collected datasets, and the responses of lettuce growth, the daily growth rate of the lettuce represents the accumulation of plant weight over time. The growth rate observed after analysing the fresh and dry weights of the lettuce plants, measured the sensitivity of the change in biomass to the change in environmental conditions, over time. This means, the dynamic effect of the environmental conditions on the lettuce growth weight can be identified more clearly by using a growth rate variable, rather than using the normal change in the growth. Thus, the daily growth rate of lettuce weight was chosen as the main output for the developed BNN modelling and prediction model.

**Figure 5:**
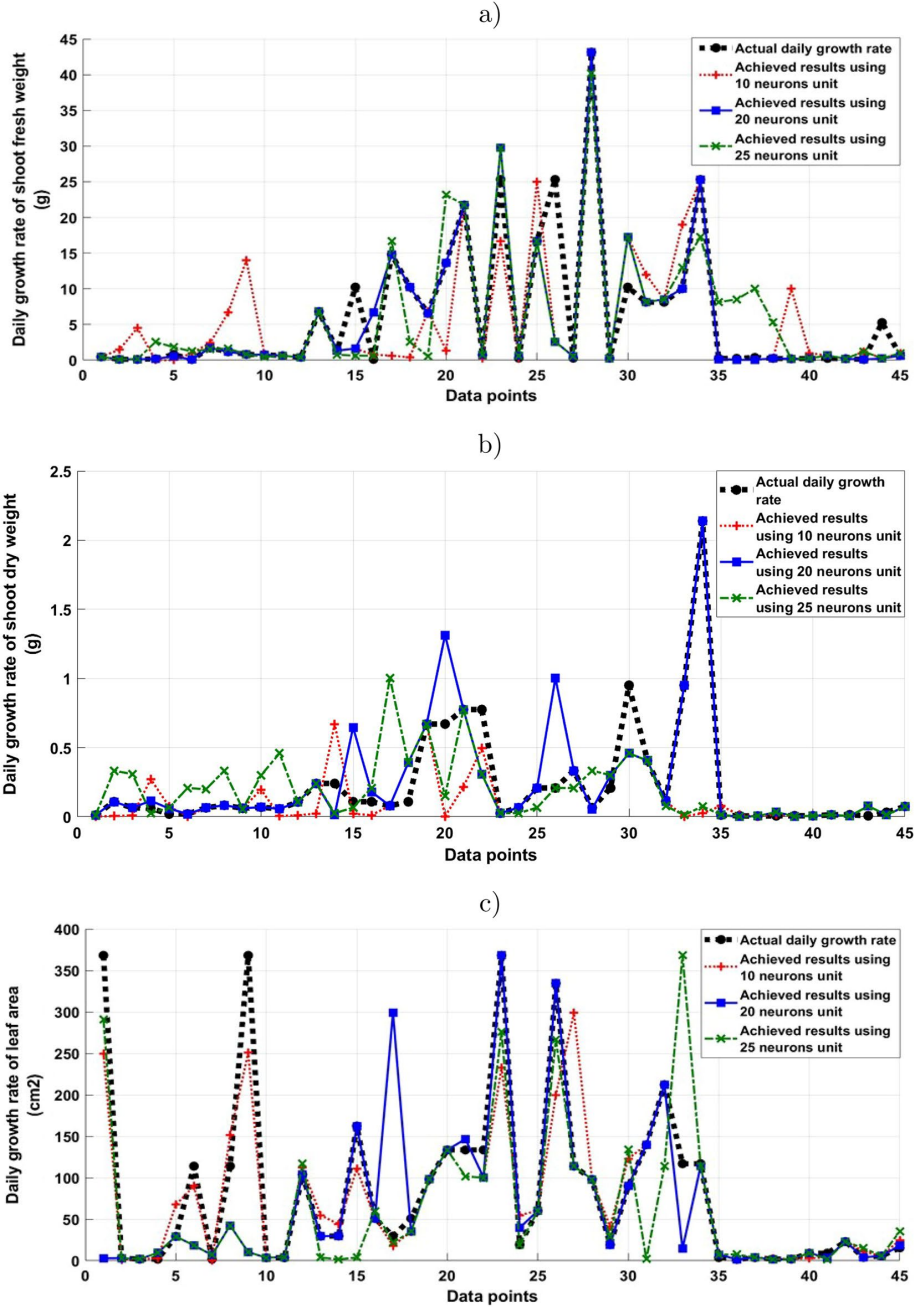
The results of the BNN models performance based on the number of neuron units in the hidden layer for predicting the daily growth rate; (**a**) shoot fresh weight, (**b**) shoot dry weight, (**c**) leaf area in the CSG.

### Evaluation of the environment benefits of CSG

#### Temperature

Temperature has a profound effect on crop growth, with every 1 $^{\circ }$C divergence from the optimum growing season mean daily temperature was found to greatly influence both head yield and cultivation time of lettuce grown in a moderate climate^12^. As would be expected due to the geographical location of the trial and time of year, the average temperature varied greatly between experiment 1 in the spring and experiment 2 in the early winter, with average outside temperatures of 17.2 $^{\circ }$C and $-7.11\,^{\circ }$C, respectively. The average temperature within the CSG was 22.96 $^{\circ }$C during the “warm” experiment and 10.9 $^{\circ }$C in the “cold” experiment, with the temperature difference inside the CSG relative to the outside environment in the warm season being 5.7 $^{\circ }$C and 18 $^{\circ }$C in the winter. The 18 $^{\circ }$C difference in average temperature inside the CSG compared to the out outside temperature show that the CSG is capable of providing an effective passive insulating and heating system during harsh winter conditions. Furthermore, comparison of the average daily minimum temperature in the winter experiment show the coldest temperature reached was $-0.98\,^{\circ }$C in the CSG compared to $-23\,^{\circ }$C outside, a difference of 22.02 $^{\circ }$C Critically, these results show that the thermal insulation and passive heating of the CSG is reducing the crop exposure to cold stress which would drastically increase productivity and profitability.

#### Light

Light is a critical environmental variable as photosynthetically active radiation is absorbed by plants to drive photosynthetic carbon fixation needed to fuel plant growth. A 58.8% seasonal difference in average light radiation was recorded in the outside environment in the winter relative to the late spring experiment. In the winter conditions, the average light radiation inside the CSG were 179.5 W/m${^2}$ in experiment 1 and 63.88 W/m${^2}$ in experiment 2. According to^27^, light was a limiting factor below 130 W/m${^2}$ on the yield of lettuce grown in a controlled environment under optimal temperatures, indicative that light could be a limiting factor between the two experiments but especially in the warm experiment which had more favourable growing conditions. The percentage light lost between inside compared to outside the CSG was 26% in the late spring and 36% in the winter, likely explained by the seasonal difference solar elevation angle affecting transmission of light through the south roof.

#### CO_2_

CO_2_ concentration was also measured as it is an important environmental factor elevated CO_2_ causes increased photosynthesis in plants through increasing the efficiency of carbon fixation, which leads to greater production of carbohydrates and biomass. The average CO_2_ concentration inside the CSG was substantially elevated in the winter compared to the spring experiment by 144 ppm. In the winter, to retain heat windows and vents are generally closed which reduces the ventilation in the glasshouse and can lead to elevated CO_2_ caused by respiration from workers and soil respiration. Previous research from has shown CO_2_ concentration generally increases crop yield, however, this was dependent on light radiation, since both CO_2_ and light be limiting factors inhibiting photosynthesis and therefore plant growth^28^. Therefore, increased CO_2_ during the winter may improve photosynthetic performance, plant growth and yield. Given the large variation between environmental factors in the two experiments, this should provide a robust dataset for accurately modelling lettuce growth in CSG.

#### Humidity

Humidity was also measured as stomata tend to close in dry air to reduce water loss which indirectly affects photosynthesis and therefore biomass accumulation due to reduced intracellular CO_2_ concentrations lowering the efficiency of photosynthetic carbon fixation. Average humidity did not vary greatly seasonally as measured from outside the CSG, with 51.3% in the late spring compared to 58.7% in th winter. In the warm experiment as the CSG is well ventilated during this time, the inside RH was only marginally increased to 51.3%. However, in the winter, when windows are closed to reduced heat loss, the inside RH was elevated to 81.75%, a 39% increase compared to the outside average RH. Slightly increased RH in the winter will improve the water use efficiency by reducing the evaporative water demand on the stomate while also improving photosynthetic performance^29^.

### Evaluation of seasonal difference in plant performance

The plant phenotypic traits measured throughout both experiment 1 and 2 are presented in Fig. 4. Cultivation time between experiments differed greatly between experiment 1 and 2, with time to harvest 35 days in the warm season and 60 days during the winter. Shoot fresh weight, which is equivalent to yield in lettuce as the entire above-ground plant material is harvested, was 320.8g per head in the winter after 60 growing days compared to 258.9g in the spring experiment after 35 days. If the yield of the crop is considered relative to the harvest time then the warm season head a greater fresh weight per day compared to the winter season, with 7.4g per day compared to 5.3g per day in the winter season, a 38% increase. Shoot dry weight, is a biomass measurement that indicates the net primary production and growth rate of the plant excluding difference in water content. Dry shoot weight was also higher in the winter experiment, with 13.8g compared to 11.8g in the warm experiment. However, if compensating for cultivation time the rate of dry weight at harvest normalised for growing time was increased by 47% in the warm season relative to the winter season. The leaf area at harvest was 3085cm${^2}$ in the warm experiment compared to 6545cm${^2}$ in the winter experiment. While the CSG offers substantial passive thermal heating to improve the growing conditions relative to the outside environment, during the winter the mean daily temperature and light intensity fall well below ideal range as reported by other studies^12,27^. The sub-optimal growing conditions in the winter necessitate an increased cultivation time which reduces the number of crop cycles which can be obtained in annually, decreasing the productivity of the CSG and leaving the potential for improvement.

**Figure 6:**
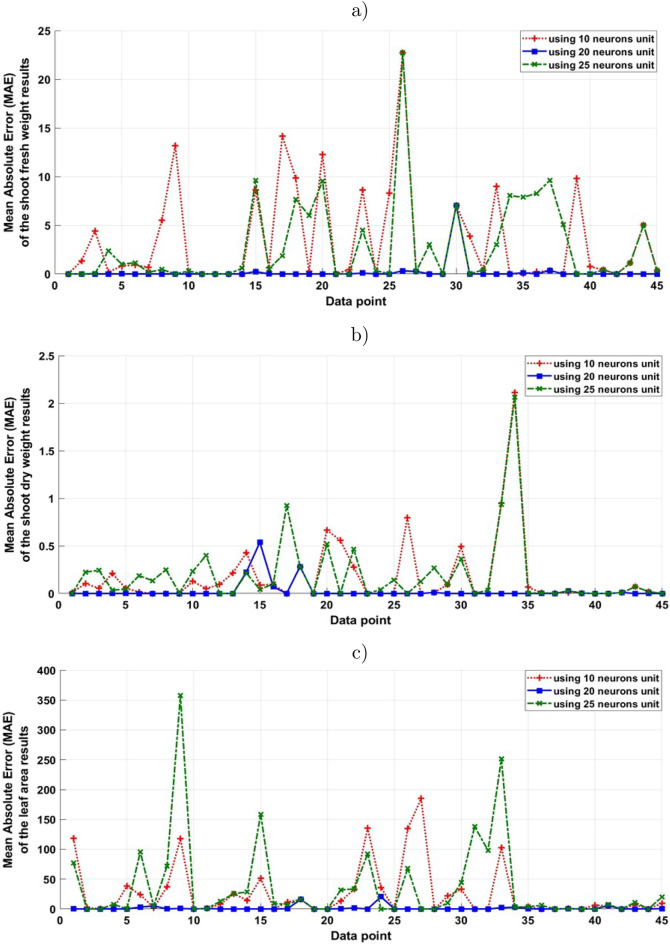
The mean absolute error values for the achieved results of using 10, 20 and 25 neuron units in the hidden for predicting the daily growth rate of (**a**) shoot fresh weight, (**b**) shoot dry weight, (**c**) leaf area in the CSG.

### Modelling daily plant growth response to environmental conditions using BNN

The lettuce growth performance in CSG, as determined by the environmental conditions, was then identified by using different structures of back-propagation neural network algorithm that employed Bayesian inference framework for modelling and predicting the lettuce fresh and dry weights growth rate was developed. The performance of the used BNN models was evaluated by comparing the predicted values (BNN models outputs) with the actual observed values (target). Figure 5, shows the comparison of the predicted estimated dynamic response of the fresh and dry weights and the leaf area that was calculated by using three different structures of BNN models and the actual observed response for the daily growth rate of the lettuce plant. The used three different structures for the number of neuron units in the hidden layer are 10, 20 and 25 neurons.

Figure 5 illustrates the difference between the predicted estimated dynamic response of the fresh and dry weights and the leaf area that was modelled by using three different structures of BNN models and the actual observed response for the daily growth rate of the lettuce plant to find the optimum required number of neurons to predict the daily plant grown in CSG. The three different structures for the number of neuron units used in the hidden layer are 10, 20 and 25 neurons. The diversity of modelled and predicted outputs of the monitored plant response parameters are different in each BNN structure once they were tested and evaluated using validation and test datasets. Lettuce shoot fresh and dry weights and leaf area are considered to observe the ability of the designed BNN models for modelling and predicting the growth response in this research study. There is a minor difference between the actual observed response with the predicted results when the BNN with 20 neuron units in the hidden layer was employed for predicting plant biomass (dry weight) and fresh weight, which indicates the high accuracy of this BNN structure is used to model and predict the plant biomass CSG. However, when the same designed BNN model was employed to model and predict the leaf area, the accuracy of the achieved result was a bit less compared with the results achieved for predicting the plant dry and fresh weights. These achieved results demonstrate the remarkable capability of the BNN to model and predict the diversity of plant growth responses. It is be seen that the BNN with 20 neuron units in the hidden layer design is followed the trend of the actual plant response parameters and showed its ability for modelling and predicting the temporal nature of the given data compared to the 10 and 25 neuron units in the hidden layer designs.

Mean absolute error rate is calculated for the obtained results from the three different used BNN structures to be compared with the observed response for the daily growth rate of plant response as it is shown in Fig. 6. In this evaluation technique, *n* is the number of errors in the observation samples, $x_i$ and *x* are the targets and the BNN achieved results values. Evaluation of the best fitting BNN designed network to optimise the most accurate BNN model structure was done by calculating the MAE values. This evaluation aims to maximise the coefficient of determination and minimise the MAE values. To minimise the MAE and achieve accurate modelling and predicting results, three different structures of back-propagation neural network algorithms that employed a Bayesian inference framework were used during the training phase for training the models.

The optimal BNN model structure is determined by the model parameters, which are a combination of environmental condition inputs (temperature, CO_2_, relative humidity and light radiation) and the number of neuron units in the hidden layer to find the best performance of the identified model. The effect of these combined input parameters was investigated. This effect is determined in Fig. 5 by calculating the mean absolute error (MAE). As it is shown in Table 2, it was found that the MAE is reached to its minimal value by 0.18, 0.02 and 1.32 when 20 neuron units are used in the hidden layer of the BNN for predicating the shoot fresh and dry weights and leaf area, respectively. Therefore, the foregoing suggests that the neural network structure with 20 neuron units in the hidden layer h is useful for modelling and predicting such a dataset.

**Table 2:** Mean absolute error values of the used BNN models with different number of hidden layers h for modelling the daily growth rate of fresh and dry weight, and the leaf area daily growth.

Shoot fresh weight			Shoot dry weight			Leaf area		
h = 10	h = 20	h = 25	h = 10	h = 20	h = 25	h = 10	h = 20	h = 25
3.36	0.18	2.85	0.17	0.02	0.18	26.58	1.32	37.60

Prediction of plant growth via BNN and other ML techniques could perform better due to its ability to handle unseen and high random data^30^. Subsequently reducing farm costs, energy use, and potential environmental damage. Incorporating IoT big data with machine learning techniques can deliver profitable, accurate and reliable outcomes, such as plant recognition and crop type classification, detection of plant and leaf diseases, fruit counting and forecasting soil moisture content^31^. Saravi et al.^32^ employed a DL technique for modelling crop yield using different weather scenarios and varying environmental variables combined with random irrigation applications to create 10,000,000 possible scenarios. Results showed that a simpler Bayesian-based DNN model with a structure of 10 neurons in 5 layers performed just as well (78.6% accuracy) when examining crop productivity, comparable to a DNN crop model with 400 neurons in 10 layers, despite the size of the neural network reducing 80-fold. Whilst machine taught crop models are becoming more extensively used to predict crop productivity, input requirements and biomass yield, existing models are complex, requiring thousands of variables to produce accurate results. DL modelling techniques and Bayesian methods present an opportunity to remedy these limitations. Khan et al.^28^ used three different DNN methods to analyse and predict the production output of major fruits based on data taken from the National Bureau of Statistics of Pakistan. The study found the Bayesian regularisation back propagation (BR) method (76.3% accuracy) to be most efficient—the Levenberg-Marquardt optimisation method (LM) and the scale conjugate gradient back propagation (SCG) method achieved 65.6% and 70.2% accuracy, respectively. Successfully adopting cross-disciplinary integration between the application of big data technology, IoT, DL techniques and our agricultural production systems is crucial for Agriculture 4.0 development. Widespread problems experienced in the agricultural field encompass crop diseases, poor pesticide control, inefficient irrigation, and ineffective weed management: all could be better controlled and remedied through automized farming practices. For food security, using such techniques for the prediction of crop yield and food availability on a national level would be influential for agricultural policy and assist in market forecasting. Additionally, AI investment can strongly influence the attainment of Sustainable Development Goals, particularly in emerging economies where a component of poverty reduction can be achieved through revolutionizing agriculture education^33^.

In this research, three different structures, in terms of the number of neurons in the hidden layer, of the BNN approach for improving modelling and prediction daily crop yield growth performance using data gathered from CSG based on sensory devices. Considering the results obtained from the conducted experiments, it can be concluded that the 20 neurons in the hidden layer model exhibit a high score for accuracy and the minimum mean absolute error (MAE) when its performance is tested for predicting the daily growth performance of the shoot fresh and dry weights and leaf area separately. Also, the overall modelling growth performance, when it is over the whole system, demonstrates the effectiveness of the proposed approaches. The BNN model shows more robust and reliable performance once applied to a larger dataset that (e.g., dataset A and B) represent warm and cold seasons. In particular, when this dataset is mixed randomly together. Our findings show that DL approaches can accurately predict plant performance using environmental factors in CSGs. Modelling crop yield for CSGs offers the potential to develop better management strategies to maximise performance and profitability, allowing economic analysis of the benefit of supplemental heating, lighting, or CO_2_ enrichment.

## Conclusions and future work

This paper confirms that the Chinese Solar Greenhouse (CSG) design is an energy-saving and low-cost design technology that combines solar energy input and appropriate heat sinks. This design allows the CSG to provide a better crop growth environment, especially in the winter, which significantly influences the greenhouse microclimate and enhances crop productivity and sustainability. It can be concluded from this paper that the Bayesian Neural Networks (BNNs) are effective in modelling and predicting plant growth in response to the temperature, CO_2_, humidity, and light radiation conditions in CSG across cold and warm seasons.

Future work can be undertaken to empirically compare this paper’s results with an array of scenarios across different growing environments and crop varieties to conclude the best intelligent crop simulation models and algorithms. This future research direction will potentially improve yield performance estimation and achieve energy-saving modelling strategies.

## Data Availability

The datasets used and/or analysed during the current study available from the corresponding author on reasonable request.
